# Gut Microbiota and Gut–Brain Axis in Hypertension: Implications for Kidney and Cardiovascular Health—A Narrative Review

**DOI:** 10.3390/nu16234079

**Published:** 2024-11-27

**Authors:** Ewelina Młynarska, Jakub Wasiak, Agata Gajewska, Aleksandra Bilińska, Greta Steć, Joanna Jasińska, Jacek Rysz, Beata Franczyk

**Affiliations:** 1 Department of Nephrocardiology, Medical University of Lodz, ul. Zeromskiego 113, 90-549 Lodz, Poland; 2 Department of Nephrology, Hypertension and Family Medicine, Medical University of Lodz, ul. Zeromskiego 113, 90-549 Lodz, Poland

**Keywords:** microbiota, hypertension, gut–brain axis, chronic kidney disease, cardiovascular health

## Abstract

Introduction: Arterial hypertension is a major contributor to a wide range of health complications, with cardiac hypertrophy and chronic kidney disease being among the most prevalent. Consequently, novel strategies for the treatment and prevention of hypertension are actively being explored. Recent research has highlighted a potential link between hypertension and the gut–brain axis. A bidirectional communication between the microbiota and the brain via the vagus nerve, enteric nervous system, hypothalamus–pituitary–adrenal axis, secreted short-chain fatty acids, and neurotransmitter metabolism. Materials and methods: A comprehensive literature search was conducted using databases such as PubMed to identify studies exploring the relationship between gut microbiota and hypertension, along with the effects of dietary interventions and probiotics on blood pressure regulation. Discussion: Studies in both animal models and human subjects have demonstrated a strong correlation between alterations in gut microbiota composition and the development of hypertension. By influencing blood pressure, the gut microbiota can potentially affect the progression of cardiovascular and kidney disorders. Modulating gut microbiota through dietary interventions and probiotics has shown promise in regulating blood pressure and reducing systemic inflammation, offering a novel approach to managing hypertension. Diets such as the Mediterranean diet, which is rich in polyphenols and omega-3 fatty acids and low in sodium, promote the growth of beneficial gut bacteria that support cardiovascular health. Additionally, probiotics have been found to enhance gut barrier function, reduce inflammation, and modulate the Renin–Angiotensin System, all of which contribute to lowering blood pressure. Conclusions: Further research is needed to determine the mechanisms of action of the microbiota in hypertension. The aim of this study was to evaluate the influence of gut microbiota on blood pressure regulation and the progression of hypertension-related complications, such as cardiovascular and kidney disorders.

## 1. Introduction

Hypertension, commonly referred to as elevated blood pressure (BP), is a chronic condition characterized by persistently increased arterial BP [1]. Guidelines recommend for hypertension to be diagnosed during repeated examination in the medical facility when the patient’s systolic blood pressure (SBP) is ≥140 mm Hg and/or their diastolic blood pressure (DBP) is ≥90 mm [2,3]. Ambulatory BP measurement (ABPM) is an out-of-office technique that allows for BP to be automatically measured, usually over a 24 h period at preselected intervals [4]. Hypertension can be diagnosed when the 24 h mean SBP is ≥130 mmHg and/or DBP is ≥80 mmHg. Furthermore, hypertension can be confirmed when the awake (daytime) SBP is ≥135 mmHg and/or DBP ≥85 mmHg and nighttime (sleep) SBP is ≥120 mmHg and/or DBP ≥70 [5].

Hypertension remains a growing health and social burden, affecting an estimated 1.56 billion adults, with its prevalence projected to reach 60% of adults worldwide by 2025 [6]. It is recognized as the largest contributor to the global disease burden, with its increasing prevalence attributed to aging populations, sedentary lifestyles, and poor dietary choices [7,8]. According to the World Health Organization (WHO), the occurrence of uncontrolled hypertension reached 26% in 2019, failing to meet the voluntary global target of 21% [9].

**Figure 1:**
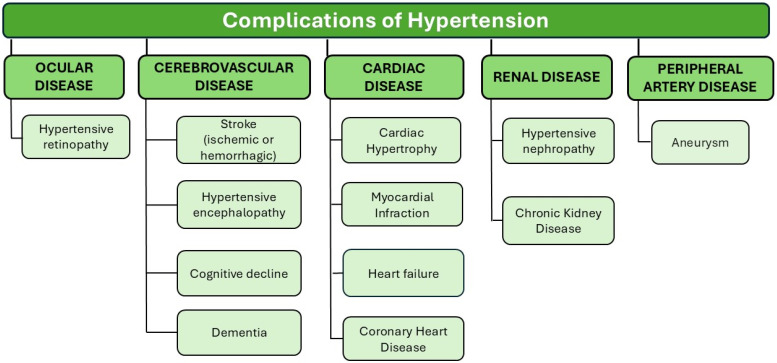
Complications of hypertension.

Globally, untreated or suboptimally controlled hypertension remains one of the leading causes of coronary heart disease [10,11]. Elevated BP is the cause of other cardiac complications, presented in Figure 1 [12,13]. Chronic BP elevation is associated with cerebral vasculature atherosclerosis and endothelial dysfunction, which can lead to neurological complications, summarized in Figure 1 [14,15]. Moreover, hypertensive nephropathy is a frequent finding in patients with hypertension, potentially leading to chronic kidney disease (CKD) [16]. Chronically elevated BP is also related to ophthalmological complications such as hypertensive retinopathy which can potentially lead to vision loss [17]. A summary of the complications of hypertension is depicted in Figure 1.

The pathophysiology of hypertension is complex and multifactorial, involving many interrelated mechanisms. The classic pathophysiology mechanism of hypertension involves dysregulation of the Renin–Angiotensin–Aldosterone System (RAAS), overactivity of the Sympathetic Nervous System (SNS), endothelial dysfunction, dysregulation of BP renal regulation, and disruption of neurohumoral factors [18]. Recent research has also highlighted the role of chronic inflammation and immune system activation in the pathogenesis of hypertension, as immune cells release pro-inflammatory cytokines which exacerbate endothelial dysfunction [19].

These interconnected mechanisms offer therapeutic targets for hypertension management, with most current drugs targeting currently well-known mechanisms. The rising health burden pushes for the development of new hypertension management strategies [20]. Recently, a connection between gut microbiota and hypertension has been proposed with a mechanism through which the host’s microbiome regulates the development and progress of hypertension, allowing for a new path in hypertension management [21,22].

The microbiota refers to microorganisms, including commensal bacteria, that reside in the human gastrointestinal tract. It is estimated that a single individual’s gut microbiome contains more than 100 trillion bacteria, increasing progressively from the stomach to the intestines and colon with around 10^12–14^ bacteria per gram of tissue [23,24]. These microbes possess genetic material 100 times greater than the human genome, and their total mass within the body is thought to range between 1 and 3 kg [25,26]. The human gut microbiota and host maintain a symbiotic connection, in which both benefit from each other. The host offers a habitat and nourishment for the microorganisms, which in return support the host’s health by enhancing disease resistance and improving nutrient absorption from digested food [23]. This balanced, cooperative state is known as eubiosis, in contrast to dysbiosis, which refers to an imbalance in the composition and function of the intestinal microbiota [27]. Due to the presence of the microbiota–gut–brain axis (GBA), which facilitates bidirectional communication between the human gut microbiota and itself, eubiosis is essential for human functioning [28]. The GBA can be characterized as interactions that involve the gut-associated immune system, the enteric nervous system (ENS), the vagus nerve, and the gut microbiota that secretes neurotransmitters, tryptophan, or short-chain fatty acids (SCFAs) [29,30,31].

The aim of this study was to comprehensively evaluate the influence of gut microbiota on blood pressure regulation and the progression of hypertension-related complications, including cardiovascular diseases and kidney disorders. Research aims to explore the potential mechanisms underlying these relationships, such as the modulation of inflammatory responses, hormonal regulation, and the production of microbiota-derived metabolites. Ultimately, this investigation aspires to provide insights that could inform novel therapeutic strategies for managing hypertension and its associated complications.

## 2. Materials and Methods

### 2.1. Literature Search Strategy

A comprehensive literature search was conducted using databases such as PubMed. The search focused on studies examining the relationship between gut microbiota and hypertension, as well as the impact of dietary interventions and probiotics on blood pressure regulation. Efforts were made to include the most recent studies to ensure up-to-date findings. Keywords used in the search included “hypertension”, “gut microbiota”, “gut-brain axis”, “probiotics”, “diet”, “blood pressure”, “cardiovascular”, and “kidney”. Both animal and human studies were considered to provide a broad understanding of the topic.

### 2.2. Inclusion and Exclusion Criteria

Inclusion Criteria: Studies were included if they

1. Investigated the association between gut microbiota composition and hypertension;
2. Evaluated the effects of dietary interventions (e.g., Mediterranean diet) or probiotics on blood pressure regulation;
3. Discussed the mechanisms of the gut–brain axis in relation to hypertension;
4. Included both animal and human studies to provide a comprehensive understanding of the topic.

Exclusion Criteria: Studies were excluded if they

1. Focused on non-hypertensive populations or did not assess blood pressure outcomes;
2. Lacked a clear link to gut microbiota or failed to provide relevant mechanistic insights.

### 2.3. Data Analysis

In this review study, data were extracted from selected studies to assess the relationship between gut microbiota and hypertension. We concentrated on key information, including sample size, study design, and population characteristics, as well as specific details of dietary interventions and the types and dosages of probiotic strains employed, along with outcomes related to blood pressure and other relevant cardiovascular health indicators. This review aimed to synthesize the findings from the included studies, highlighting the role of the gut–brain axis and the mechanisms connecting the gut microbiota to hypertension and hypertension-related complications. Additionally, this review aimed to identify gaps in the current literature and suggest future research directions to further explore these important connections.

## 3. Microbiota and the Gut–Brain Axis

The microbiota’s composition is unique to each person and changes over time, reaching a relatively stable state during adulthood. The microbiota begins forming in the fetal stage, and the method of birth—whether vaginal or cesarean—plays a key role in its early development. Additionally, an infant’s diet, particularly whether they are breastfed or formula-fed, significantly influences the composition of their microbiota [32,33]. The microbiota’s composition is distinct, yet in healthy people, the proportions are relatively consistent. Typically, *Firmicutes* account for 60% to 80% of the total, *Bacteroidetes* make up 20% to 40%, while *Proteobacteria* and *Actinobacteria* each represent about 5%. Research shows that 20% of microbiota diversity can be attributed to short-term dietary changes; however, long-term changes in diet have a greater influence on microbiota composition [34,35,36]. We distinguish three main enterotypes of microbiota depending on the predominance of bacteria genera, as shown in Table 1 [37,38]. Overall, other factors that affect the type and number of bacteria present in the gut are environmental pollution, medications and antibiotics taken, place of residence, and origin [39,40]. Medical conditions also significantly influence microbiota composition, with abnormalities noted in cases of in type 2 diabetes, gastroenterological diseases (irritable bowel syndrome, chronic constipation, and gastroesophageal reflux disease), hypertension, cardiovascular diseases, chronic kidney disease, and psychiatric disorders, among others [29,41,42,43,44,45,46,47,48].

**Figure 2:**
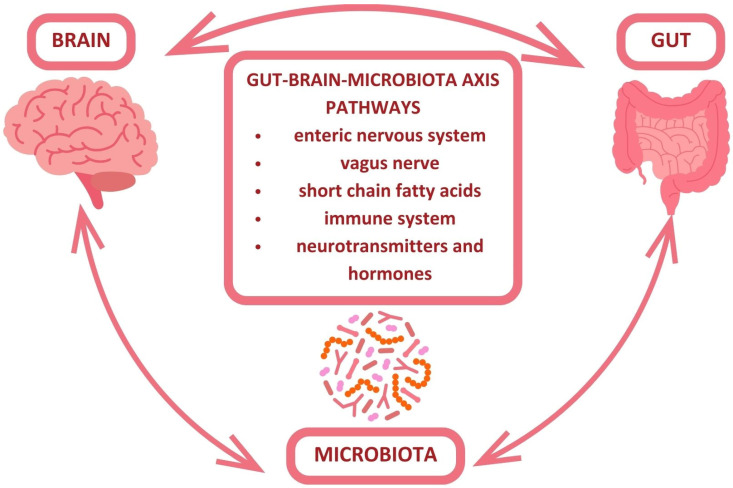
Gut–brain–microbiota axis pathways simplified.

As previously mentioned, the GBA facilitates bidirectional communication between the human gut microbiota and itself, and its main pathways are presented in Figure 2 [29,30,31].

The autonomic nervous system, especially through the vagus nerve, along with the ENS, made of enteric neurons and glial cells, play key roles in regulating gut secretion, motility, and immune responses. This allows the body to influence the gut microbiota’s composition [49,50]. In turn, gut microbes can communicate with the central nervous system (CNS) through neuronal, hormonal, and immune pathways, impacting various bodily functions. SCFAs, like butonate and propionate, produced by gut bacteria from fiber fermentation, can regulate human gene expression via histone hyperacetylation. SCFAs also exert systemic effects, including immunomodulation, appetite control, calcium absorption, and glucose regulation [51,52,53,54]. Additionally, the gut microbiota influences CNS activity through alterations in the production and metabolism of neurotransmitters and molecules such as acetylcholine, catecholamines, histamine, adenosine, and tryptophan [55,56,57]. For instance, norepinephrine slows overall transit and lowers the number of migratory motor complexes in the gut. Furthermore, it has anti-inflammatory properties and has a role in behavior and cognition, including learning, memory, and attention [55,56]. Adenosine similarly exhibits local anti-inflammatory and immunomodulatory effects [58].

The proper functioning of the GBA relies on the integrity of the intestinal barrier, which separates the inner environment of the intestinal lumen from the rest of the human body, thereby limiting solute and fluid exchange between the lumen and tissues [59]. When dysbiosis occurs, barrier function is compromised, causing increased intestinal permeability, commonly referred to as “leaky gut” syndrome (LGS). LGS is associated with the translocation of microbial components such as lipopolysaccharide (LPS), toxic metabolites, and inflammatory agents into the circulatory system. In the body, LPS is detected by Toll-like receptors (TLRs), especially TLR4. Bacterial toxins also activate nuclear factor κB, which, together with TLR4, regulates the expression of cytokines. This activation triggers the release of pro-inflammatory molecules and interleukins (IL), including tumor necrosis factor-alpha (TNF-α), IL-6, IL-8, and IL-12, resulting in both local and systemic inflammation [60,61,62]. Interestingly, the loss of ENS cells may induce increased intestinal permeability even if there is no inflammation in the tissue [63]. On the other hand, Polysaccharide A, a microbial-derived substance produced by Bacteroides fragilis, is detected by TLR2, which initiates a protective anti-inflammatory response in the CNS [64].

The assessment of intestinal barrier function in LGS is performed by using a multi-sugar test involving five different sugar probes: sucrose, lactulose, L-rhamnose, erythritol, and sucralose. However, novel approaches for assessing permeability have recently emerged, focusing on blood biomarkers such as LPS, lipopolysaccharide-binding protein (LBP), intestinal fatty acid-binding protein (I-FABP), zonulin, and calprotectin [29].

The chronic, systemic inflammation associated with LGS disrupts the hypothalamus–pituitary–adrenal (HPA) axis, crucial for providing high-energy fuels like glucose, amino acids, and free fatty acids to support immune responses. This dysregulation leads to increased glucocorticoid and catecholamine release and triggers hypercortisolemia and overactivity of the HPA axis, which is associated with impaired glucocorticoid receptor function [65]. Moreover, in the case of increased intestinal permeability, T-cell activation can occur. This can lead to the development of autoimmune disorders in the gut or other organs if activated lymphocytes are transmitted further [66].

Additionally, low-grade systemic inflammation in LGS impairs the brain–blood barrier (BBB), which is critical for maintaining brain homeostasis and normal neuronal function by restricting the passage of chemicals, ions, and cells into the brain [67,68]. Increased BBB permeability reduces the quantity and function of astrocytes and increases the activity of microglia [69,70]. Research indicates that these alterations increase the risk of developing psychiatric disorders, including depression and Alzheimer’s disease [62,71].

As previously noted, dysbiosis, LGS, and GBA dysfunction are common in various disease states. The mechanisms behind these associations are complex and still being studied. However, they are linked to bacterial translocation, low-grade inflammation, and microbiota-derived substances. Their role will be discussed in more detail later in this article.

## 4. Alterations in Gut Microbiota Composition in Hypertension

### 4.1. Hypertension and Gut Microbiota in Animal Models

In recent years, multiple studies on humans and various animal models have demonstrated a strong correlation between hypertension and gut microbiota.

One of the first studies to suggest the influence of microbial composition on BP regulation was conducted by Mell et al. Using the Dahl rat model, which includes both salt-sensitive (S) and salt-resistant (R) strains on a high-salt diet (HSD), they observed significant differences in the microbial content of S and R rats [72]. The microbiota of S rats was characterized by an increased prevalence of bacteria from the phylum *Bacteroidetes*, particularly with the greater abundance of the family S24-7 within this phylum, as well as the higher abundance of the family *Veillonellaceae* from the phylum *Firmicutes*, compared to R rats. Another study on the Dahl salt-sensitive rats found a positive association between BP and the abundance of six taxa, including taxa of the *Pseudomonadales* order, the *Christensenellaceae*, *Barnesiellaceae*, *Eubacteriaceae* families, and the *Erwinia* and *Anaerofustis* genera. Conversely, the abundance of the *Anaerostipes* genus displayed a negative correlation with BP. Moreover, the microbial profile of the HSD-fed animals contained an increased number of taxa from the genus *Erwinia* and the families *Christensenellaceae* and *Corynebacteriaceae* and a lower number of taxa from the genus *Anaerostipes* compared to the control group fed with regular chow [73]. In a murine model, high salt intake led to the depletion of *Lactobacillus species* [74]. Treatment with *Lactobacillus* spp. supplementation in HSD-fed mice diminished salt-induced hypertension, presumably by the modulation of the activity of pro-inflammatory Th17 cells [74].

Studies on another genetic model of spontaneously hypertensive rats also revealed differences in the composition of gut microbiota compared to normotensive controls, including reduced microbial richness and diversity, a lower number of acetate- and butyrate-producing bacteria, and an increased Firmicutes-to-Bacteroidetes ratio [75,76].

A close relationship between dysbiosis and BP regulation has been confirmed in two other models of laboratory-induced hypertension: the deoxycorticosterone acetate (DOCA)-salt model and angiotensin II (Ang II)-induced hypertension. In hypertensive DOCA-salt rats, an increase in the Bacilli class of *Firmicutes* and *Lactobacillales* family was detected, while the bacteria genera of *Sutterella*, *Actinobacteria*, and *Oscillospira* were observed to be reduced [77]. Using germ-free (GF) mice, Karbach et al. assessed the role of intestinal microbiota in the pathogenesis of Ang II-induced hypertension, along with its effects on inflammatory markers and resulting organ damage [78]. They evidenced that treatment with Ang II resulted in increased BP in both researched groups; however, the effect was notably muted in GF mice compared to the conventional animals. Furthermore, the GF mice were protected from vascular oxidative stress and inflammation and showed less cardiac fibrosis and immune cell infiltration in the kidneys, suggesting the involvement of microbiota in these pathological processes. In related research, Yang et al. demonstrated alterations in gut microbiota composition following Ang II infusion, observing a notable decrease in microbial richness and an elevated Firmicutes-to-Bacteroidetes ratio compared to the control group [75]. Subsequent treatment with minocycline, an anti-inflammatory antibiotic, was able to alter the microbiota composition by reducing the Firmicutes-to-Bacteroidetes ratio, simultaneously attenuating high blood pressure and suggesting a connection between these two processes [75].

In summary, the findings from all experimental models, as presented in Table 2, showed that hypertensive animals exhibit altered microbiota compared to their normotensive counterparts, suggesting a complex yet undeniable interplay between microbiota and the diverse pathophysiological mechanisms underlying hypertension.

### 4.2. Microbiota and Hypertension in Clinical Studies

Numerous clinical studies have established a strong association between dysbiosis and hypertension, as shown in Table 3. In a 2017 study by Li et al., the analysis of the metabolome and metagenome in a cohort of primarily hypertensive, pre-hypertensive, and healthy patients revealed a significant decrease in microbial richness and diversity among hypertensive patients, along with a distinct metagenomic profile, including an overgrowth of bacteria such as *Prevotella* and *Klebsiella* [79]. Interestingly, the microbiome characteristics in pre-hypertensive patients closely resembled those in hypertensive individuals. Subsequent fecal transplantation of the microbiota from hypertensive human donors to germ-free mice resulted in the elevation of blood pressure in the recipient animals, suggesting a direct role of microbiota in BP regulation [79]. This finding was later supported by the large cohort study Coronary Artery Risk Development in Young Adults (CARDIA), which confirmed an inverse correlation between gut microbial diversity and both hypertension and systolic BP [80]. More specifically, the data revealed a correlation between hypertension and 18 different genera, including *Anaerovorax*, *Clostridium IV*, *Oscillibacter*, and *Sporobacter*, and the distribution of *Veillonelnotably* aligned with hypertensive patients. In addition, *Anaerovorax*, *Catabacter*, and *Robinsoneilla* have been positively correlated with hypertension [80]. In a cohort study on 6953 Finnish participants, Palmu et al. observed 45 microbial genera positively associated with BP, 27 of which belonged to the phylum *Firmicutes* [81]. In another study, the intestinal microbiota of patients with hypertension has been identified by an elevated quantity of opportunistic pathogens, such as *Klebsiella*, *Streptococcus*, and *Parabacteroides* [82]. In contrast, the number of SCFA producers, including *Roseburia* and *Bacillus freundii*, was notably reduced.

Significantly, certain bacteria have been suggested to influence blood pressure fluctuations. A positive correlation was observed between greater variability and the abundance of *Clostridium* and *Prevotella*, while lower variability was associated with higher levels of *Alistipes finegoldii* and *Lactobacillus* [83].

## 5. Impact on Kidney and Cardiovascular Health

**Figure 3:**
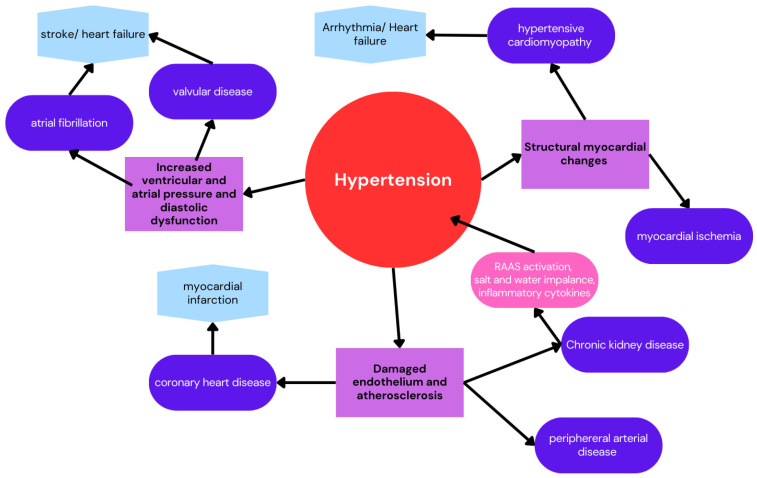
Hypertension results on CVD.

Hypertension is one of the most important risk factors for cardiovascular disease (CVD) and CKD. The cardiovascular consequences of high blood pressure are extensive and include atrial fibrillation, valvular heart disease, cardiomyopathy, coronary artery disease, peripheral arterial disease, and chronic kidney disease, as well as stroke, heart failure, and cardiovascular death. Each condition underscores the critical importance of managing blood pressure to reduce the risk of severe health outcomes [84]. A summary of various mechanisms leading to CVD and CKD is shown in Figure 3.

The microbiome, acting as a mediator of oxidative stress and chronic inflammation, also contributes to the pathophysiology and risk of developing CVD [85].

**Figure 4:**
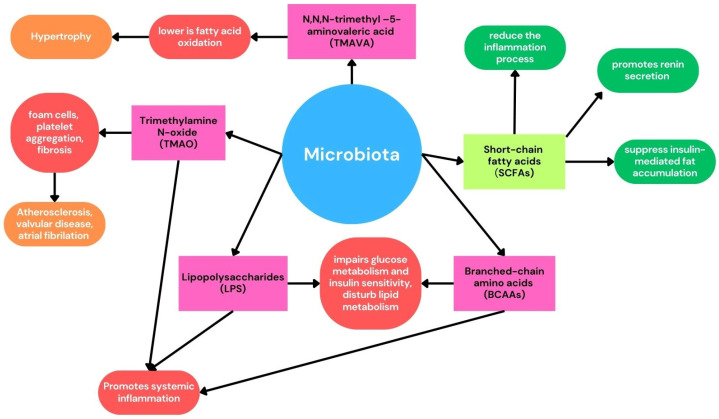
Microbiota products and their actions.

Several mechanisms may be responsible for this, with one of the most critical being the binding of lipopolysaccharide to TLR4, causing the secretion of pro-inflammatory molecules and enhancement of pro-atherogenic receptors. This mechanism has been shown to contribute to atherosclerosis, atrial fibrillation, and heart failure. Other microbiota-derived products, such as trimethylamine N-oxide (TMAO) and bile acids, also influence the progression of CVD. These molecules are linked to increased inflammatory states promoting atherogenesis, fibrosis, and foam cell formation. Conversely, SCFAs inhibit inflammation and play a cardioprotective role [86]. Their roles and mechanisms are illustrated in Figure 4 and discussed in further detail later in this article.

### 5.1. Damaged Endothelium and Atherosclerosis

Atherosclerosis is the buildup of plaques within arterial walls, leading to serious complications that affect not only the cardiovascular system but also other organs. It can cause coronary artery disease, reducing blood flow to the heart muscle and potentially leading to ischemia and myocardial infarction. Plaques in other arteries may lead to peripheral artery disease, strokes, or chronic kidney disease. Hypertension adds mechanical stress to the artery walls, damaging the endothelium over time and promoting the inflammatory process, which is a key factor in plaque buildup. This process results in atherosclerosis [87]. Hypertension is also linked to the activation of the RAAS and Sympathetic Nervous System, promoting the release of pro-inflammatory cytokines (e.g., IL-6, IL-1β, TNF-alpha) from vascular cells [88].

Microbiota changes also play an important role in the development of atherosclerosis, acting not only as a modulator of the inflammation process and oxidative stress but also through various different mechanisms. SCFAs can activate G-protein-coupled receptors such as GPR41 and GPR43, reducing the inflammation process and influencing enteroendocrine regulation to suppress insulin-mediated fat accumulation [89]. They also affect renin secretion and modulate blood pressure. Therefore, SCFAs lower the overall risk of CVD. On the other hand, there are microbiota products such as branched-chain amino acids (BCAAs) and LPS that influence glucose metabolism and insulin sensitivity [48]. They also cause disturbed lipid metabolism and contribute to atherosclerotic plaque formation [90].

Some nutrients are metabolized by gut bacteria into trimethylamine (TMA), which the liver then oxidizes to Trimethylamine N-oxide. TMAO promotes the accumulation of cholesterol in macrophages, thus fostering the formation of foam cells and atherosclerosis. Additionally, TMAO alters platelet calcium signaling and enhances platelet aggregation, contributing to endothelial dysfunction and CVD [91,92,93].

Gut bacteria are responsible for primary bile acids’ conversion and therefore for lipid metabolism, glucose homeostasis, and inflammatory processes. Dysregulation in bile acid metabolism can contribute to cardiovascular disease by promoting inflammation, altering lipid profiles, and impacting vascular function [94,95]. Additionally, gut microbiota may be a source of bacteria that accumulates in the arteriosclerotic plaque and may impact plaque stability and the development of other cardiovascular conditions [89,96]. It is suggested that symptomatic atherosclerosis is associated with different microbiota genus and that the bacterial metagenome may contribute to development of this disease. The presence of bacteria such as *Collinsella* may be more typical for individuals affected by symptomatic atherosclerosis [97].

### 5.2. Structural Myocardial Changes

Increased blood pressure forces the left heart chamber to work harder to pump blood to the rest of the body. Over time, this additional strain activates intracellular signaling cascades and the synthesis of actin and myosin that compose the sarcomeres, added parallelly in the cardiomyocyte and increasing its size [98].

This condition, known as left ventricular hypertrophy (LVH), leads to structural changes in the heart, fibrosis, and reduced blood flow to the heart muscle. It impairs the heart’s ability to function properly, increasing the risk of congestive heart failure or myocardial infarction [98].

Microbiota also adds to this process: the higher amount of N, N,N-trimethyl–5-aminovaleric acid (TMAVA) produced by gut bacteria, the lower is fatty acid oxidation, and higher the risk of cardiac hypertrophy [99,100].

However, animal model experiments do not always support the theory that diet may influence hypertrophy. One study found that when a probiotic composed of *Lactobacillus plantarum* was added to the rat’s diet, the process of developing LVH and heart failure after myocardial infarction was attenuated [101]. On the other hand, an experiment examining the impact of a high-fiber and high-acetate diet showed no preventative effect, despite these factors being significant modulators of gut microbiota that release SCFAs. The diet did not prevent cardiac remodeling, hypertrophy, or overall dysfunction in animals with dilated cardiomyopathy [102].

### 5.3. Increased Ventricular and Atrial Pressure and Diastolic Dysfunction

Mitral regurgitation (MR), one of the most prevalent valvular heart diseases, involves the retrograde flow of blood from the left ventricle (LV) into the left atrium due to improper closure of the mitral valve during systole. [103]. It can be caused by structural deformities such as degenerative changes in the leaflets or damage that prevent the valve leaflets from closing properly during systole. Chronic volume overload from MR can lead to left atrial and ventricular dilation, adversely affecting ventricular filling and relaxation. Hypertension exacerbates these effects by increasing myocardial wall stress, contributing to left ventricular remodeling and worsening MR. Conditions such as ischemic cardiomyopathy or left ventricular remodeling, such as dilated cardiomyopathy, can also cause mitral regurgitation. The stiffening of the ischemic ventricle requires higher filling pressures to maintain adequate preload, which in turn increases pressure in the left atrium and leads to mitral regurgitation. In dilated cardiomyopathy, the enlargement of the left ventricle further exacerbates the issue by impairing the proper closure of the mitral valve, contributing to the regurgitation and potentially leading to progressive heart failure [104].

Gut dysbiosis causes hypertension, contributes to LVH, and therefore also links to valvular diseases. Together with environmental factors, it can lead to chronic, subclinical inflammation in the body. This inflammation promotes the infiltration of inflammatory cells into the heart valves, which, along with pro-inflammatory molecules, triggers a cascade of reactions that cause the valves to calcify, become fibrotic, and eventually malfunction [86]. TMAO is proven to influence valve fibrosis by initiating endoplasmic reticulum stress mechanisms involving the activation of PERK/ATF-4 and IRE-1α/XBP-1s pathways, which may particularly affect the aortic valves [105]. Therefore, hypertension and changes in gut microbiota may contribute to the development of mitral regurgitation, aortic stenosis, or aortic regurgitation.

The combination of increased left atrial pressure and volume leads to atrial dilation. This process promotes fibrosis and structural remodeling, which disrupts the normal conduction pathways within the atria, predisposing to rhythm disorders such as atrial fibrillation (AF). Studies also suggest a link between gut microbiota and AF. TMAO has been associated with an increased risk of AF. Additionally, certain bacterial genera, such as *Enorma* and *Bifidobacterium*, have been associated with both the prevalent and incidence forms of AF [106].

### 5.4. Hypertension and Chronic Kidney Disease

CKD is characterized by a gradual loss of kidney function over time, and it is a precursor to end-stage renal disease. It is also associated with an increased risk of cardiovascular disease. Hypertension, which is a huge risk factor for CVD, may be encountered during the progression of kidney disease and is also a leading cause of its progression [107].

There are several interconnected factors influencing both of those diseases, including volume expansion, the RAAS, oxidative stress, inflammatory states, endothelial dysfunction, and vascular remodeling [107].

Diabetes leads to endothelial damage and increased cytokine secretion [108] and is a strong predictor of incident CKD and the rapid decline of kidney function [109]. Dyslipidemia and oxidative stress, present in inflammation, contribute to atherosclerosis. Vessel thickening, a reduction in the availability of small blood vessels in the kidney, and decreased perfusion can lead to a decline in renal blood flow (RBF), glomerular filtration rate (GFR), and tubular function [110]. This reduces the ability to accurately filter waste and fluid from the blood. When sodium is not filtered and excreted properly, the extracellular expansion provokes hypertension and edema [107].

The RAAS, which controls the water and salt homeostasis in the human organism, is highly influenced by hypertension and kidney disease. The synthesis of renin is stimulated when renal scarring occurs [111]. It may also be stimulated by oxidative stress, gut microbiota dysbiosis, and nitric oxide deficiency [112], which are present in various CVD. The activated RAAS contributes to both systemic and glomerular capillary hypertension [113], a vicious cycle where proper blood pressure and renal function become interdependent.

## 6. Gut Microbiota and Hypertension: Clinical Implications and Future Perspectives for Kidney and Cardiovascular Health

Recent research has greatly advanced our understanding of the intricate role of gut microbiota in hypertension and its implications for kidney and cardiovascular health. Understanding this dynamic interplay is crucial as it offers new avenues for prevention, diagnosis, and treatment of hypertension-related complications. This chapter explores future research directions, clinical implications, and current insights into how gut microbiota affects hypertension and, consequently, overall health.

### 6.1. Microbiome Profiling: A New Approach to Hypertension Prevention

Integrating gut microbiota screening for individuals at high risk of hypertension may significantly enhance early detection and prevention efforts. Research suggests that specific microbial patterns are associated with hypertension, such as a reduced diversity of beneficial bacteria (*Lactobacillus* and *Bifidobacterium*) and an increase in potentially harmful bacteria (*Firmicutes* and *Proteobacteria*). Identifying gut microbiota patterns may aid in early identification of individuals at risk for hypertension. Integrating these microbiota profiles into risk assessments may enable clinicians to more accurately predict hypertension susceptibility, potentially allowing for targeted interventions for those at higher risk.

Microbiota profiles could also support personalized dietary recommendations designed to restore a healthy gut microbiome. Additionally, lifestyle modifications, including physical activity and stress management, could be tailored based on microbiota findings to further support gut health and blood pressure regulation.

### 6.2. Advancing Hypertension Management with Microbiota-Focused Interventions

Integrating gut microbiota modulation into standard hypertension treatment protocols offers a promising enhancement to therapeutic outcomes. Dietary interventions play a key role in both managing hypertension and modulating gut microbiota. By tailoring dietary plans to individual gut microbiota profiles, it is possible to significantly influence blood pressure regulation.

The Mediterranean and Dietary Approaches to Stop Hypertension (DASH) diets are renowned for their cardiovascular benefits and their positive impact on gut microbiota composition. These diets emphasize the consumption of polyphenol-rich foods, omega-3 fatty acids, and reduced sodium, which together contribute to both lowering blood pressure and enhancing cardiac health [114,115]. Polyphenols found in foods such as berries, olive oil, and nuts regulate the gut microbiota by being metabolized into SCFAs [116]. SCFAs, in turn, increase beneficial bacteria like *Bifidobacteria* and *Lactobacilli* while suppressing harmful ones like Clostridium [117], reducing systemic inflammation and supporting endothelial function, which helps lower blood pressure [116,118]. Omega-3 fatty acids, found in fatty fish, seeds, and nuts, enhance the growth of beneficial bacteria such as *Lactobacillus* and *Akkermansia muciniphila*, improve gut barrier function [119], and may reduce pro-inflammatory cytokines like TNF-α, IL-6, and IL-1β by increasing SCFA production [120,121]. A reduced sodium intake contributes to blood pressure regulation by decreasing fluid retention and easing cardiovascular stress [122]. Although sodium’s direct effects on the gut microbiota are less studied, high dietary sodium consumption can lead to gut dysbiosis by altering the composition and diversity of gut microbiota and negatively affecting intestinal permeability. This disruption increases the production of inflammatory cytokines, which can, in turn, directly elevate blood pressure [123].

A meta-analysis conducted by Zhao et al. examined the effects of long-term (≥8 weeks) probiotic use on office and ambulatory BP. The meta-analysis included 26 trials with a total of 1624 participants, demonstrating that probiotic consumption significantly reduced office systolic BP by 2.18 mmHg and diastolic BP by 1.07 mmHg. Additionally, analysis of ambulatory BP from three trials showed a similar reduction of −2.35 mmHg in systolic BP and −1.61 mmHg in diastolic BP. Subgroup analyses revealed that hypertensive and diabetic patients experienced significant reductions in both systolic and diastolic BP (*p* ≤ 0.02). Interestingly, the study found that factors such as increasing age, baseline body mass index (BMI), treatment duration, and baseline systolic BP did not lead to significant enhancements in the antihypertensive effects of probiotics [124].

Specific probiotic strains, notably *Lactobacillus* and *Bifidobacterium*, have shown promising antihypertensive effects [125]. These probiotics may help regulate blood pressure through several key mechanisms. They enhance gut barrier function by increasing the production of tight junction proteins, such as occludin and claudin, which prevents the translocation of inflammatory molecules from the gut into the bloodstream. This process reduces systemic inflammation, a known contributor to hypertension [126]. The studies show that probiotics may inhibit pro-inflammatory cytokines like IL-1, IL-6, and TNF-α and produce anti-inflammatory cytokines like IL-4, IL-10, IL-11, and IL-13, reducing the overall inflammatory state in the body, which may help in lowering blood pressure [127]. Additionally, probiotics modulate the RAAS, which plays a crucial role in blood pressure regulation. For instance, *Lactobacillus helveticus* produces bioactive peptides during protein fermentation, which can inhibit angiotensin-converting enzyme (ACE), reducing the production of angiotensin II, a potent vasoconstrictor [128,129]. This action may contribute to blood pressure reduction. Probiotic supplementation has also been shown in many studies to reduce total cholesterol, LDL-C, and triglycerides and increase HDL-C, consequently improving vascular health and potentially lowering blood pressure [130].

Potentially, another microbiota-targeted intervention may be fecal microbiota transplantation (FMT). Xu et al. studied the effects of FMT on blood pressure in spontaneous hypertensive rats (SHRs) in comparison to normotensive Wistar Kyoto (WKY) rats. Their research involved transplanting fecal microbiota from WKY rats to SHRs and vice versa, measuring blood pressure and analyzing gut microbial composition through 16S rDNA gene amplicon sequencing. The results indicated that after FMT, blood pressure decreased in SHRs and increased in WKY rats. Significant differences in gut microbial composition were observed, particularly in the abundance of lactic and butyric acid-producing bacteria. Furthermore, FMT influenced intestinal mucosal barrier integrity, affecting the expression of tight junction proteins and indicating altered mucosal permeability [131].

There are also human studies focusing on microbiota transplantation which further support the potential of this intervention in managing hypertension. In a study conducted by Zhong, patients who underwent washed microbiota transplantation (WMT) demonstrated a reduction in both systolic and diastolic blood pressure. Specifically, systolic blood pressure decreased by −5.09 ± 15.51 mmHg (*p* = 0.009), while diastolic blood pressure showed a reduction of −7.74 ± 10.42 mmHg (*p* < 0.001). Additionally, the study identified factors influencing the antihypertensive response to WMT. Patients who underwent the procedure via the lower gastrointestinal tract experienced a greater reduction in systolic blood pressure. Notably, those not taking antihypertensive medications also demonstrated a more pronounced decrease in both systolic and diastolic blood pressure [132]. Given these results, FMT may represent a novel strategy for addressing hypertension through microbiota modulation, paving the way for future research into its clinical applications in human populations.

Well-designed clinical trials are crucial to determine the most effective probiotic strains and their optimal dosages for different patient populations. These trials can help establish standardized guidelines for using probiotics as part of hypertension management and provide insights into the long-term safety and efficacy of probiotic supplementation, ensuring that these treatments can be tailored to individual needs and integrated into comprehensive care plans.

Moreover, lifestyle changes may significantly impact hypertension management by enhancing gut microbiota balance and overall health. For example, a study showed that moderate exercise led to a significant increase in SCFAs and lactic acid producers, also increasing the relative abundance of *Akkermansia muciniphila*, which is associated with improved metabolic health and reduced inflammation [133,134]. Additionally, stress management is crucial for maintaining gut microbiota balance, as chronic stress can lead to dysbiosis and increase intestinal permeability [29]. Such changes in gut health are associated with systemic inflammation, which can disturb blood pressure regulation [135]. Mind–body interventions, including yoga and meditation, may potentially counteract these effects by reducing stress-induced cortisol secretion [136], thereby supporting gut microbiome stability and improving gastrointestinal barrier function.

### 6.3. Future Directions in Microbiota-Based Hypertension Interventions

Regular gut microbiota screening could enable the continuous monitoring of microbial composition changes in response to interventions, helping to evaluate the effectiveness of personalized treatments and facilitate adjustments as needed. For example, if initial interventions do not produce the desired results, clinicians might modify dietary or probiotic strategies based on updated microbiota profiles. Furthermore, integrating gut microbiota screening and targeted therapies with conventional hypertension treatments could optimize antihypertensive medication choices and potentially reduce side effects. Enhancing gut health may improve the efficacy of medications or allow for reduced dosages.

Ongoing research will be crucial to clarify the precise mechanisms through which gut microbiota influences blood pressure. Large-scale longitudinal studies may be necessary to validate the efficacy of microbiota-based interventions and assess their long-term effects on hypertension. Future research should prioritize clinical trials to identify the most effective strains and dosages for different patient populations, ensuring that probiotic interventions are both safe and efficacious. Such studies will help refine treatment protocols and integrate probiotic therapies into standard hypertension management practices.

Advances in microbiome analysis technologies and data interpretation are likely to continue refining personalized treatment strategies. As research advances, the development of novel biomarkers and diagnostic tools based on gut microbiota profiles may become feasible. These tools could facilitate the early detection of individuals at risk for hypertension and guide the implementation of personalized, microbiota-targeted interventions.

## 7. Conclusions

Emerging evidence suggests that gut microbiota may play a significant role in regulating blood pressure. Although initial studies are promising, the findings remain preliminary and require further investigation. Dietary adjustments, the use of probiotics, and other lifestyle modifications have shown potential benefits, but these interventions need to be validated through larger and more rigorous studies. Future research should focus on confirming the specific mechanisms by which gut microbiota influence blood pressure, refining and optimizing treatment protocols based on these insights and integrating microbiota-based strategies into clinical practice. This comprehensive approach could potentially enhance hypertension management and provide more personalized treatment options.

## Figures and Tables

**Table 1:** Enterotypes of microbiota in humans.

Enterotype	Abundant Bacteria Species	Type of Diet
1	*Bacterioides*	high-fat diet and low fiber intake, industrialized food
2	*Prevotella*	high fiber intake, low meat and dairy intake
3	*Ruminococcus*	high-fat and -protein diet

**Table 2:** Composition of intestinal microbiota in hypertensive animal models. HSD—high-salt diet, SHR—spontaneously hypertensive rats, DOCA—deoxycorticosterone acetate, Ang II—angiotensin II, ⭡— increase ⭣— decrease.

Reference	Model	Intestinal Microbiota Alterations in Hypertensive Animals
Mell et al. [72]	HSD-fed rats	⭡ *Bacteroidetes*, *Veillonellaceae*,
Bier et al. [73]	HSD-fed rats	⭡ *Pseudomonadales*, *Christensenellaceae*, *Barnesiellaceae*, *Eubacteriaceae*, *Erwinia*, *Anaerofustis* ⭣ *Anaerostipes*
Wilck et al. [74]	HSD-fed rats	⭣ *Lactobacillus*
Yang et al. [75] Adnan et al. [76]	SHR	⭣ microbial richness and diversity ⭣ acetate- and butyrate-producing bacteria ⭡ *Firmicutes-to-Bacteroidetes* ratio
Robles-Vera et al. [77]	DOCA-salt rats	⭡ *Firmicutes*, *Lactobacillales* ⭣ *Sutterella*, *Actinobacteria*, *Oscillospira*
Yang et al. [75]	Ang II-induced hypertension	⭣ microbial richness ⭡ *Firmicutes-to-Bacteroidetes* ratio

**Table 3:** Composition of intestinal microbiota in hypertensive patients. ⭡— increase ⭣— decrease.

Reference	Population	Intestinal Microbiota Alterations in Hypertensive Patients
Li et al. [79]	99 hypertensions 56 pre-hypertensions 41 controls	⭡ *Prevotella*, *Klebsiella* ⭣ microbial richness and diversity
Sun et al. [80]	529 subjects (183 hypertensions)	⭡ *Anaerovorax*, *Clostridium IV*, *Oscillibacter*, *Sporobacter*, *Catabacter*, *Robinsoneilla*
Palmu et al. [81]	6953 subjects	⭡ *Firmicutes*
Yan et al. [82]	60 hypertensions 60 controls	⭡ *Klebsiella*, *Streptococcus*, *Parabacteroides* ⭣ *Roseburia*, *Bacillus freundii*

## Data Availability

The data presented in this study are available upon request from the corresponding author due to the partial obtainment of articles within time limits. For full-version access, contact the corresponding author.

## Abbreviations

BP—blood pressure; SBP—systolic blood pressure; DBP—diastolic blood pressure; ABPM—ambulatory blood pressure measurement; WHO—World Health Organization; CKD—chronic kidney disease; RAAS—Renin–Angiotensin–Aldosterone System; SNS—Sympathetic Nervous System; GBA—gut–brain axis; ENS—enteric nervous system; SCFAs—short-chain fatty acids; CNS—central nervous system; LGS—leaky gut syndrome; LPS—lipopolysaccharide; TLRs—Toll-like receptors; IL—interleukin; TNF-α—tumor necrosis factor-alpha; LBP—lipopolysaccharide-binding protein; I-FABP—intestinal fatty acid-binding protein; HPA—hypothalamus–pituitary–adrenal; HSD—high-salt diet; SHR—spontaneously hypertensive rats; DOCA—deoxycorticosterone acetate; Ang II—angiotensin II; CVD—cardiovascular diseases; TMAO—trimethylamine N-oxide; BCAAs—branched-chain amino acids; TMA—trimethylamine; LVH—left ventricular hypertrophy; TMAVA—N, N,N-trimethyl–5-aminovaleric acid; AF—atrial fibrillation; RBF—renal blood flow; GFR—glomerular filtration rate; DASH—Dietary Approaches to Stop Hypertension; BMI—body mass index; ACE—angiotensin-converting enzyme; FMT—fecal microbiota transplant.
